# Natural Ventilation for Prevention of Airborne Contagion: Authors' Reply

**DOI:** 10.1371/journal.pmed.0040195

**Published:** 2007-05-29

**Authors:** Adrian Roderick Escombe, David A. J Moore, Jon S Friedland, Carlton A Evans, Robert H Gilman

We would like to thank the correspondents for their thoughtful contributions to this important public health topic [1]. As our abstract and article state, we measured natural and mechanical ventilation and then calculated the effects of these ventilation rates on estimated tuberculosis (TB) infection rates using a mathematical model of airborne infection. This appears to be the first published assessment of natural ventilation rates in health-care settings, and the novel conclusions of our article are that extremely high rates of dilutional ventilation can be achieved through natural ventilation at very little cost by simply opening windows and doors. Indeed, this natural ventilation was far in excess of even the best maintained mechanical ventilation systems used in health-care settings. Importantly, this natural ventilation greatly reduced the calculated risk of airborne infection.

Measuring TB transmission itself is difficult, as rates in staff are confounded by exposures outside the workplace, and mechanical air sampling techniques have had limited success. We have established a guinea pig air sampling facility to directly measure TB transmission in a hospital ward in Lima, Peru [2] and have used this model to evaluate the effects of upper room ultraviolet light and negative air ionization on TB transmission. We plan to use this facility to further study natural ventilation, and its effect on actual TB transmission.

The results of the current study cannot be generalized to regions too cold to tolerate enhanced natural ventilation and not every room may be as amenable to natural ventilation as the Peruvian rooms that we studied. However, the key conclusions are clear: high rates of natural ventilation were achieved even on days with little wind and even rooms without high ceilings and large windows were well ventilated, such that natural ventilation significantly exceeded mechanical ventilation.

It is therefore clear that natural ventilation has an important role to play in the fight against institutional TB transmission in resource-limited settings. Mechanical ventilation is expensive to install, requires costly ongoing maintenance, may be dangerous if poorly maintained (for example, delivering positive instead of negative pressure), and is clearly inappropriate for the great majority of resource-limited settings where the burden of TB is highest. TB infection control is an urgent priority, underscored by the emergence of extreme drug-resistant TB strains and the increasing congregation in potentially high-risk overcrowded settings of persons living with HIV through the roll-out of enhanced HIV care. When infectious TB patients share rooms with others, opening windows and doors to enhance natural ventilation is a simple, inexpensive, and effective strategy in the fight against nosocomial TB transmission.
